# Coordinated Ecophysiological Trait Shifts of *Populus euphratica* Along a Groundwater-Depth Gradient: From Carbon Acquisition Toward Water Conservation in an Arid Riparian Forest

**DOI:** 10.3390/plants15091295

**Published:** 2026-04-22

**Authors:** Yong Zhu, Hongmeng Feng, Ran Liu, Jie Ma, Xinying Wang

**Affiliations:** 1 Xinjiang Institute of Ecology and Geography, Chinese Academy of Sciences, Urumqi 830011, China; zhuyong23@mails.ucas.ac.cn (Y.Z.); fenghongmeng23@mails.ucas.ac.cn (H.F.); liuran@ms.xjb.ac.cn (R.L.); 2 Fukang Desert Ecology National Field Scientific Observation and Research Station, Chinese Academy of Sciences, Fukang 831505, China; 3 University of Chinese Academy of Sciences, Beijing 100049, China; 4 Xinjiang Tarim River *Populus euphratica* Forest Ecosystem Positioning Observation and Research Station, Xinjiang Uygur Autonomous Region Forestry Academy, Urumqi 830046, China; 5 Xinjiang Field Scientific Observation and Research Station of Luntai *Populus euphratica* Forest Ecosystem, Urumqi 830046, China; 6 Xinjiang Key Laboratory of Forestry-Grassland Sand Control and Sand Industry, Urumqi 830046, China

**Keywords:** carbon-nitrogen stoichiometry, groundwater drawdown, hydraulic conductivity, leaf functional traits, stomatal anatomy, WUE_i_

## Abstract

Under the combined pressures of climate change and irrigated cropland expansion, groundwater tables are declining rapidly across arid regions, thereby intensifying water limitation in riparian ecosystems. However, the mechanisms by which dominant riparian tree species coordinate multiple functional traits to maintain carbon–water balance remains poorly understood. This study investigated coordinated ecophysiological trait shifts of *Populus euphratica* Oliv. along a groundwater-depth gradient (2.19, 4.88, and 7.45 m) in the middle reaches of the Tarim River (China), hereafter referred to as shallow, middle, and deep groundwater depths, respectively. We quantified photosynthetic, hydraulic, stomatal, leaf anatomical and nutrient traits, and estimated long-term intrinsic water-use efficiency (WUE_i_) from foliar δ^13^C. As the groundwater table declined, (1) photosynthetic capacity and photochemical performance decreased, whereas WUE_i_ increased markedly from 38.5 ± 2.9 to 54.2 ± 1.0 μmol mmol^−1^, accompanied by the lowest transpiration rate at the deep groundwater depth (4.6 ± 0.5 mmol m^−2^ s^−1^); (2) stomatal and anatomical adjustments consistent with water-loss reduction were observed, including a significant decline in stomatal density from 93.5 ± 14.5 to 79.3 ± 17.4 pores mm^−2^, and reduced stomatal size and stomatal area fraction (−20.3% and −32.7%, respectively); (3) the percentage loss of hydraulic conductivity increased, whereas sapwood-specific hydraulic conductivity declined, accompanied by greater sapwood investment relative to leaf area, with Huber value rising from 0.06 ± 0.02 to 0.11 ± 0.04 mm^2^ cm^−2^ at deep water depth; and (4) chlorophyll concentrations and leaf water content declined, whereas structural investment increased, as reflected by higher specific leaf mass and leaf dry matter content, and leaf nutrients were enriched, with total nitrogen and total phosphorus increasing by 67.1% and 42.0%, respectively. Trait-WUE_i_ relationships further indicated that WUE_i_ covaried most strongly with leaf anatomical and nutrient traits. These results demonstrate that increasing groundwater depth was associated with coordinated shifts in carbon assimilation, water-use regulation, hydraulic function, and nutrient allocation in *P. euphratica*. Such trait coordination may help explain how this species persists under chronic water limitation in arid riparian forests.

## 1. Introduction

Climate change and its consequences for ecosystem structure and function have emerged as a central focus in ecosystem ecology. Rising temperatures and shifting precipitation regimes strongly regulate plant ecophysiological processes [1,2,3]. Simultaneously, intensifying human activities-particularly the rapid expansion of irrigated croplands are reshaping regional water budgets and exacerbating water scarcity in drylands [4]. These changes have profound implications for plant water availability, photosynthesis, and growth in arid and semi-arid regions [5]. As warming intensifies and precipitation becomes more variable, plants in drylands face escalating water limitation, which degrades growth conditions and increases ecological vulnerability [6,7]. Understanding how plants in arid regions cope with water stress and regulate their carbon-water balance is therefore crucial for sustaining ecosystem stability and long-term ecological barrier function under ongoing climate change and land-use intensification [8,9,10].

The Tarim River Basin in northwestern China constitutes a typical desert-oasis ecotone. Its riparian forests are dominated by *Populus euphratica* Oliv., a foundation tree species that strongly shapes ecosystem structure and functioning by stabilizing dunes, regulating hydrological processes, buffering microclimate, sequestering carbon, and supporting biodiversity [11,12,13,14,15,16]. Yet these forests are increasingly threatened by sustained groundwater table decline driven by groundwater abstraction and land-use intensification, with impacts further exacerbated by climate change [13,17,18]. Growing evidence indicates that falling groundwater levels constrain *P. euphratica* by weakening plant water supply and root function, thereby imposing downstream limitations on photosynthesis and reshaping the carbon-water balance [19,20,21,22]. Drought stress can reduce the net photosynthetic rate and alter intercellular CO_2_ concentration through both stomatal and non-stomatal limitations. The maximum quantum yield of photosystem II (PSII) (Fv/Fm) is also sensitive to water deficit, reflecting damage to or recovery of photosystem II under changing moisture conditions [23]. In desert plants, increasing groundwater depth has been associated with reduced carbon assimilation, deeper water uptake, lower branch water potential, and decreased gas-exchange performance [24]. Water stress can also reshape plant morphology and resource allocation, including adjustments in leaf structure, root traits, and nutrient stoichiometry [25]. Mechanistic insight into how groundwater fluctuations regulate the ecophysiological functioning of *P. euphratica* is therefore crucial for informing effective conservation and restoration of desert riparian ecosystems.

Although previous studies have examined how groundwater levels influence plant physiological processes, most have focused on individual indicators rather than integrated trait responses, and comprehensive assessments linking groundwater table change to coordinated physiological and ecological traits in *P. euphratica* remain limited [26,27,28]. In this study, we testes three hypotheses:

**H1.**  *Groundwater-table decline forces P. euphratica to shift from carbon acquisition toward water conservation, reflected in reduced photosynthetic capacity and increased long-term intrinsic water use efficiency (WUEi).*

**H2.**  *This shift is mediated through coordinated adjustments in stomatal morphology, leaf anatomy, and hydraulic architecture.*

**H3.**  *Leaf nutrient enrichment under deep groundwater conditions represents a concentration effect driven by constrained leaf expansion rather than enhanced nutrient uptake.*

This study aims to provide a basis for understanding how groundwater-table decline affects the ecophysiological functioning of *P. euphratica* in desert riparian ecosystems.

## 2. Results

### 2.1. Differences in Functional Traits of Populus euphratica Under Varying Groundwater Depths

#### 2.1.1. Leaf Gas Exchange and Fluorescence Parameters

**Figure 1:**
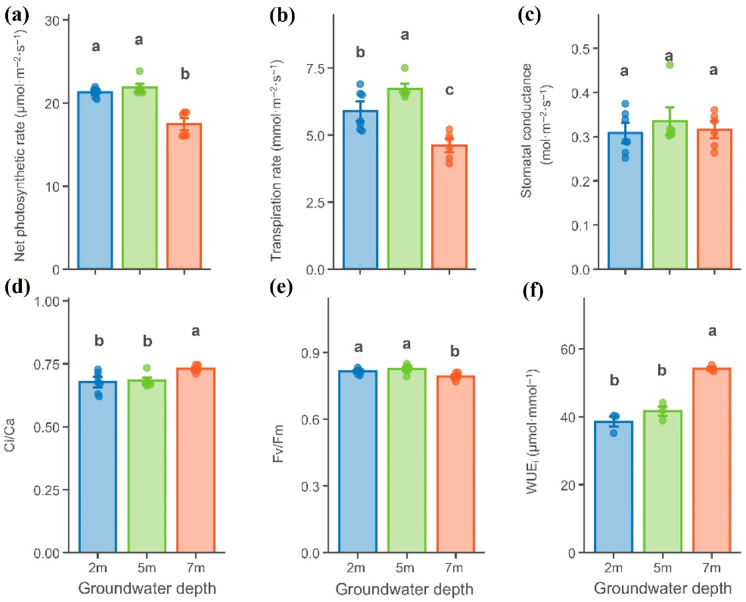
Gas exchange and chlorophyll fluorescence parameters of *Populus euphratica* at different groundwater depths. (**a**) Net photosynthetic rate; (**b**) transpiration rate; (**c**) stomatal conductance; (**d**) intercellular-to-atmospheric CO_2_ ratio; (**e**) maximum quantum yield of PSII; (**f**) long-term intrinsic water-use efficiency. Different lowercase letters denote significant differences among different groundwater depths (one-way ANOVA with LSD post hoc test, *p* < 0.05).

The net photosynthetic rate (P_n_) and maximum quantum yield of PSII (Fv/Fm) of *P. euphratica* leaves did not differ significantly between the 2 m and 5 m groundwater depth, but were significantly lower at 7 m (*p* < 0.05, Figure 1a,e). The transpiration rate (Tr) varied significantly among groundwater depths, reaching its lowest value at 7 m (4.61 ± 0.50 mmol m^−2^ s^−1^, Figure 1b). Long-term intrinsic water-use efficiency (WUE_i_) increased significantly with increasing groundwater depth, being significantly higher at 7 m (54.15 ± 1.00 μmol mmol^−1^) than at 2 m (38.54 ± 2.92 μmol mmol^−1^) and 5 m (41.63 ± 2.64 μmol mmol^−1^, Figure 1f). Similarly, the ratio of intercellular to atmospheric CO_2_ concentration (Ci/Ca) showed no significant difference between 2 m and 5 m, but increased significantly at 7 m (Figure 1d). Stomatal conductance (gs) s did not differ significantly among groundwater depths (Figure 1c).

#### 2.1.2. Leaf Stomatal Morphology and Anatomy

**Figure 2:**
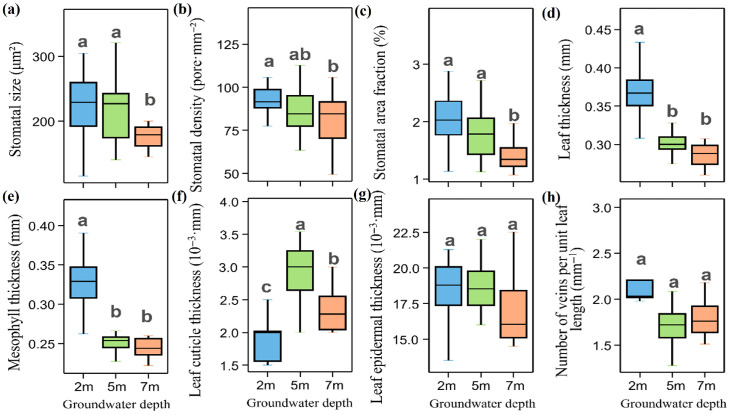
Stomatal morphology and leaf anatomical characteristics of *Populus euphratica* at different groundwater depths. (**a**) Stomatal size; (**b**) stomatal density; (**c**) stomatal area fraction; (**d**) leaf thickness; (**e**) mesophyll thickness; (**f**) leaf cuticle thickness; (**g**) leaf epidermal thickness; (**h**) number of veins per unit leaf length. Different lowercase letters denote significant differences among groundwater depths (one-way ANOVA with LSD post hoc test, *p* < 0.05).

Stomatal size (StS) and stomatal area fraction (SAF) did not differ significantly between the 2 m and 5 m groundwater depths, but both decreased significantly at 7 m (Figure 2a,c). Stomatal density (SD) differed significantly among groundwater depths, with the value at 2 m (93.47 ± 14.45 pores mm^−2^) being significantly higher than that at 7 m (79.34 ± 17.35 pores·mm^−2^, Figure 2b). Leaf thickness (LT) and mesophyll thickness (MT) were both significantly lower at the 5 m and 7 m groundwater depths than at 2 m (Figure 2d,e). Leaf cuticle thickness (LCT) varied significantly among groundwater depths, with the lowest value observed at 2 m (Figure 2g).

#### 2.1.3. Branch Hydraulic Properties

**Figure 3:**
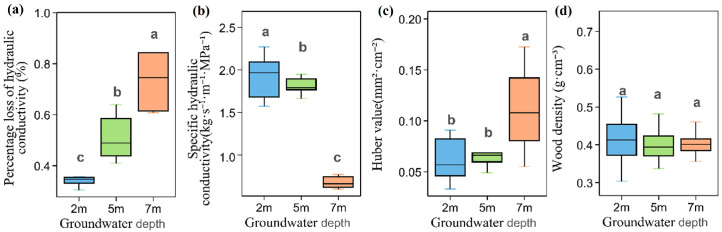
Hydraulic traits of *Populus euphratica* at different groundwater depths. (**a**) Percentage loss of hydraulic conductivity; (**b**) sapwood-specific hydraulic conductivity; (**c**) Huber value; (**d**) wood density. Different lowercase letters denote significant differences among groundwater depths (one-way ANOVA with LSD post hoc test, *p* < 0.05).

The percentage loss of hydraulic conductivity (PLC) increased significantly with increasing groundwater depth, while sapwood-specific hydraulic conductivity (Ks) declined significantly (Figure 3a,b). The Huber value (Hv) also varied significantly among groundwater depths, with the highest value observed at 7 m (0.11 ± 0.04 mm^2^ cm^−2^), which was significantly greater than those at 2 m (0.06 ± 0.02 mm^2^ cm^−2^) and 5 m (0.06 ± 0.01 mm^2^ cm^−2^, Figure 3c). Wood density (WD) showed no significant difference among groundwater depths (Figure 3d).

#### 2.1.4. Economic Spectrum Traits

With increasing groundwater depth, leaf dry matter content (LDMC) increased significantly, whereas leaf water content (LWC) declined significantly (Table 1). Specific leaf mass (SLM) was significantly higher at the 7 m groundwater depth than at 2 m and 5 m. The concentrations of chlorophyll a and chlorophyll b at a groundwater depth of 2 m (1.15 ± 0.02 and 0.29 ± 0.01 mg g^−1^, respectively) were significantly higher than those at a groundwater depth of 5 m (0.71 ± 0.01 and 0.16 ± 0.01 mg g^−1^) and a groundwater depth of 7 m (0.76 ± 0.16 and 0.20 ± 0.03 mg·g^−1^, Table 1).

**Figure 4:**
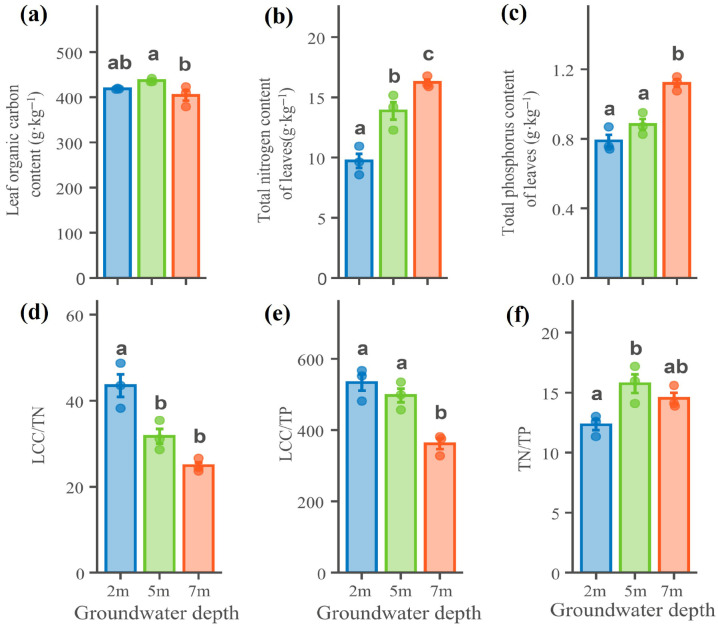
Leaf nutrient characteristics of *Populus euphratica* at different groundwater depths. (**a**) Leaf organic carbon; (**b**) total nitrogen; (**c**) total phosphorus; (**d**) carbon-to-nitrogen ratio (LCC/TN); (**e**) carbon-to-phosphorus ratio (LCC/TP); (**f**) nitrogen-to-phosphorus ratio (TN/TP). Different lowercase letters denote significant differences among groundwater depths (one-way ANOVA with LSD post hoc test) (*p* < 0.05).

Leaf nutrient contents and stoichiometric ratios of *P. euphratica* differed significantly among groundwater depths (Figure 4). At the 7 m groundwater depth, leaf total nitrogen (TN) and total phosphorus (TP)were significantly higher than at 2 m (Figure 4b,c). The carbon-to-nitrogen ratio (LCC/TN) was significantly higher at 2 m than at 5 m and 7 m. The carbon-to-phosphorus ratio (LCC/TP) also differed significantly among groundwater depths, with the value at 7 m being significantly lower than at 2 m and 5 m (Figure 4).

### 2.2. Multivariate Variation in Functional Traits Across Groundwater Depths

**Figure 5:**
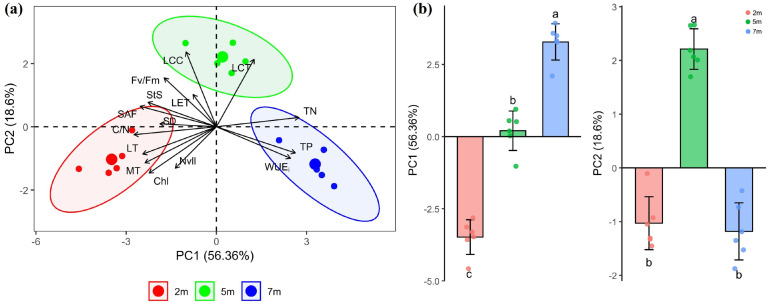
Distribution of 16 functional traits along the first two axes of principal component analysis (**a**) and the differences in axis scores among individual trees at three groundwater depths (**b**). The 16 traits are: net photosynthetic rate (P_n_), maximum quantum yield of PSII (Fv/Fm), transpiration rate (Tr), long-term intrinsic water-use efficiency (WUE_i_), stomatal size (StS), stomatal density (SD), stomatal area fraction (SAF), leaf thickness (LT), mesophyll thickness (MT), leaf cuticle thickness (LCT), specific leaf mass (SLM), leaf organic carbon (LCC), total nitrogen (TN), total phosphorus (TP), carbon-to-nitrogen ratio (C/N), and number of veins per unit leaf length (Nvll). 2 m, 5 m, and 7 m denote shallow, middle, and deep groundwater depths, respectively. Different lowercase letters denote significant differences among groundwater depths (one-way ANOVA with LSD post hoc test, *p* < 0.05).

Principal component analysis (PCA) of 16 functional traits, revealed that the first two principal components (PC1 and PC2) explained a cumulative 74.96% of the total variance (PC1 = 56.36%, PC2 = 18.6%, Figure 5a). PC1 was positively correlated with TN (0.33), TP (0.32), and WUE_i_ (0.30), and negatively correlated with C/N (−0.33), SAF (−0.31), LT (−0.30), and MT (−0.29). PC2 was positively associated with LCC (0.50), LCT (0.45), and Fv/Fm (0.33) and negatively associated with Chl (−0.31) and the number of veins per unit leaf length (Nvll; −0.28). Individual trees sampled at the shallow groundwater depth were primarily aligned with the LT and MT vectors; those at the middle depth clustered near the LCC and LCT vectors; and those at the deep depth were closely associated with WUE_i_, TN, and TP. Scores in both PC1 and PC2 differed significantly among the three groundwater depths (Figure 5).

### 2.3. Relationships Between WUE_i_ and Other Functional Traits

**Figure 6:**
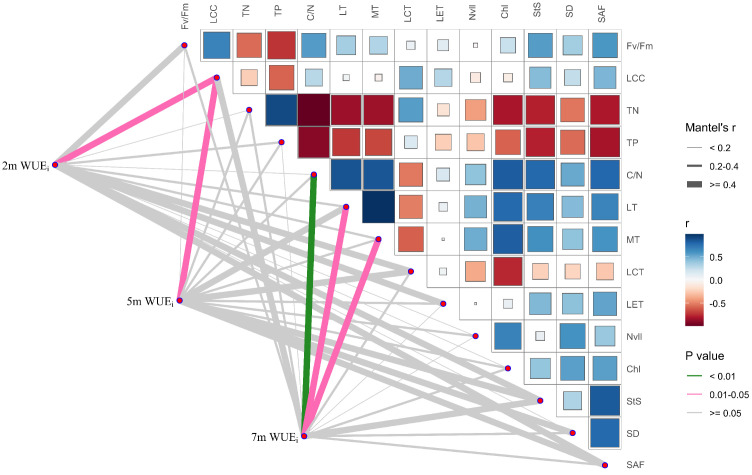
Mantel correlation and trait correlation matrix for WUE_i_ and functional traits of *Populus euphratica* at different groundwater depths.

At groundwater depths of 2 m and 5 m, WUE_i_ was significantly correlated with leaf organic carbon (LCC; *p* < 0.05, Figure 6). In contrast, at 7 m, WUE_i_ was very significantly correlated with C/N (*p* < 0.01) and significantly correlated with LT and MT (*p* < 0.05; Figure 6).

In Figure 6, the right panel shows pairwise Pearson correlation coefficients (r) among traits; color indicates the direction and strength of correlations (red = negative, blue = positive; darker colors represent larger |*r*|), and square size is proportional to |*r*|. Lines on the left represent Mantel test results between WUE_i_ and individual traits at each groundwater depth; line width denotes Mantel’s r (coupling strength), and line color indicates significance level (green, *p* < 0.01; pink, 0.01 ≤ *p* < 0.05; grey, *p* ≥ 0.05).

## 3. Discussion

**Figure 7:**
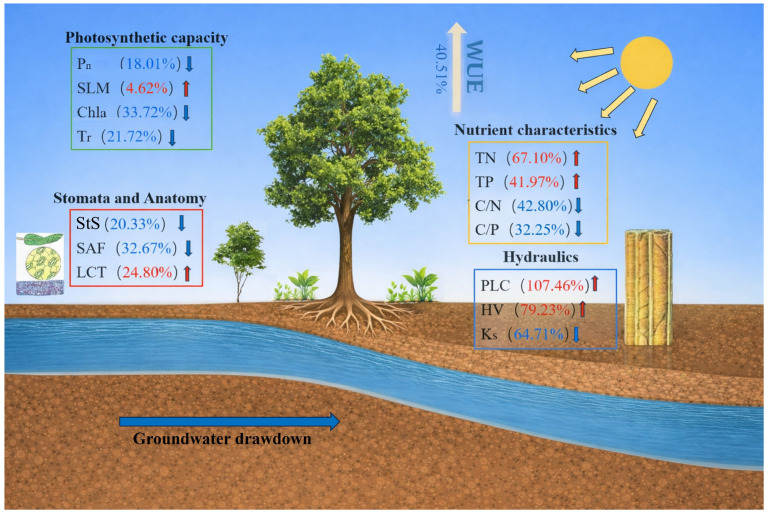
Conceptual diagram summarizing the overall shifts in functional traits of *Populus euphratica* across groundwater depths from 2 to 7 m. Upward red arrows indicate trait increases; downward blue arrows indicate decreases with increasing groundwater depth. P_n_, net photosynthetic rate; SLM, specific leaf mass; Chla, chlorophyll a; Tr, transpiration rate; WUE_i_, long-term intrinsic water-use efficiency; StS, stomatal size; SAF, stomatal area fraction; LCT, leaf cuticle thickness; TN, total nitrogen; TP, total phosphorus; C/N, leaf organic carbon-to-total nitrogen ratio; C/P, leaf organic carbon-to-total phosphorus ratio; PLC, percentage loss of hydraulic conductivity; HV, Huber value; Ks, sapwood-specific hydraulic conductivity.

This study investigated the ecophysiological responses of *P. euphratica* to varying groundwater depths in an arid riparian forest and revealed coordinated variation across multiple functional traits. Significant shifts in leaf morphology, physiology, and nutrient allocation were observed in response to groundwater drawdown. Photosynthetic capacity, stomatal size, and leaf thickness decreased, while TN, TP, and WUE_i_ increased significantly. Specifically, WUE_i_ increased by 40.51%, while stomatal size and stomatal area fraction (SAF) decreased by −20.33% and −32.67%, respectively. Additionally, the percentage loss of hydraulic conductivity (PLC) and Huber value (HV) increased by 107.46% and 79.23%, respectively, while sapwood-specific hydraulic conductivity (Ks) decreased by −64.71% (Figure 7). These shifts highlight the pattern by which *P. euphratica* optimized water-use efficiency and maintained ecological function under increasing water stress.

### 3.1. Stomatal and Anatomical Responses Under Different Groundwater Depths

With increasing groundwater depth, water availability in the habitat of *P. euphratica* declined significantly, and the leaves appeared to respond first through adaptive adjustments in stomatal morphology and anatomical structure. Stomatal size and stomatal area fraction did not differ significantly between the 2 m and 5 m groundwater depths, but both decreased significantly at 7 m. Stomatal density (SD) also declined significantly under deep groundwater conditions, with StS and SAF decreasing by 20.33% and 32.67%, respectively, from 2 m to 7 m (Figure 7). These findings indicate that under water-limited conditions, *P. euphratica* reduced water loss by decreasing stomatal size and limiting the effective area available for gas exchange. This pattern is consistent with Pan et al. [29], who report that stomatal length and width of *P. euphratica* leaves at a groundwater depth of 7.5 m were significantly smaller than those at 3.2 m and 4.2 m. Similarly, Driesen et al. [30] observe significant reductions in stomatal size in *Ocimum basilicum* L. under drought treatment.

In contrast to stomatal morphological changes, leaf cuticle thickness (LCT) reached its highest value at the intermediate groundwater depth (5 m), suggesting that the cuticular response to water stress may exhibit a threshold-like pattern. This finding aligns with the conclusion of Wei et al. [31] that the cuticle plays a key role in suppressing non-stomatal transpiration and reducing leaf water loss. Pan et al. [32] further demonstrate that the coordinated regulation of stomatal traits and photosynthetic parameters represents an important pathway by which cool-temperate plants maintain high water-use efficiency. Taken together, these results suggest that *P. euphratica* did not respond to increasing groundwater depth through changes in a single trait alone, but rather through the coordinated adjustment of stomatal morphology and leaf anatomical structure, thereby effectively controlling water consumption. These findings are consistent with hypothesis H2, supporting the role of stomatal–anatomical coordination in the adaptive response to groundwater drawdown.

### 3.2. Leaf Functional Trait Response Under Different Groundwater Depths

According to the leaf economics spectrum framework, leaf functional traits and their coupling relationships, reflect core resource trade-offs that underlie the formation and maintenance of plant economic strategies [33,34,35]. In the present study, the leaf economic spectrum traits of *P. euphratica* were significantly modified across different groundwater depths. With increasing groundwater depth, LDMC increased progressively and significantly, whereas LWC declined significantly, indicating that *P. euphratica* enhanced structural carbon investment under water-limited conditions and adopted a more conservative resource-use strategy. Specific leaf mass (SLM) was significantly higher at 7 m than at 2 m and 5 m, with no significant difference between the shallow and intermediate groundwater depths. This result suggests that *P. euphratica* substantially increased structural investment per unit leaf area only under severe water stress, further reflecting a conservative resource-use pattern consistent with the stress-tolerant end of the plant economics spectrum [36]. Reich et al. [37] likewise notes that leaves with higher SLM and LDMC generally exhibit greater drought resistance but lower photosynthetic rates.

Leaf total nitrogen and total phosphorus were significantly higher at 7 m than at 2 m and 5 m, with TN and TP increasing by 67.10% and 41.97%, respectively, from 2 m to 7 m (Figure 7). This result differs from the decline in nutrient concentrations commonly observed in short-term drought-stress experiments [38,39,40]. One possible explanation is that, under deep groundwater conditions, leaf growth and leaf area expansion were constrained, while leaf structural investment increased, leading to a concentration effect for nitrogen and phosphorus on a mass basis. Recent evidence also suggests that phosphorus may play a role in sustaining physiological performance under drought conditions [41]. In addition, as a typical deep-rooted desert tree species, *P. euphratica* is highly dependent on groundwater, and changes in groundwater depth may regulate root distribution and the uptake of deep soil water and dissolved nutrients, thereby maintaining or even enhancing leaf nutrient supply. Meanwhile, chlorophyll content declined significantly with increasing groundwater depth. This water-stress-induced reduction in chlorophyll corresponded with the lower photosynthetic rate observed at the deep groundwater depth, suggesting either impairment of the photosynthetic machinery or an adaptive reduction in photosynthetic investment under drought [42,43]. These results provide partial support for hypothesis H3, as the nutrient enrichment observed was more consistent with a concentration effect than with enhanced uptake.

### 3.3. Relationship Between WUE_i_ and Functional Traits Under Different Groundwater Depths

Long-term intrinsic water-use efficiency (WUE_i_), derived from leaf δ^13^C, increased significantly as groundwater depth increased from 2 m to 7 m. WUE_i_ at 7 m was significantly higher than that at 2 m and 5 m, representing an overall increase of 40.51% (Figure 7). This result indicates that under restricted groundwater supply, *P. euphratica* adopted a more efficient water-use pattern through functional trait adjustment to cope with prolonged drought, consistent with H1. Mantel association analysis further showed that WUE_i_ was significantly coupled with leaf carbon investment, carbon–nitrogen allocation, and leaf structural traits (Figure 6). Moreover, *P. euphratica* exhibited distinct multidimensional patterns of WUE_i_ regulation across groundwater depths. Under shallow groundwater conditions, where water availability was relatively high and water limitation was weak, variation in WUE_i_ was mainly driven by leaf organic carbon content. Higher leaf organic carbon content reflects greater accumulation of photosynthetic assimilates and structural carbon compounds, thereby increasing carbon gain per unit water consumption and maintaining WUE_i_ at a relatively low level [44]. This suggests that under favorable water conditions, *P. euphratica* prioritized stable carbon gain.

When groundwater depth increased to 7 m, water stress became markedly stronger [45], and the significant associations of WUE_i_ with the carbon-to-nitrogen ratio, leaf thickness, and mesophyll thickness suggest that the dominant trait associations underlying WUE_i_ had shifted. The significant association between WUE_i_ and C/N suggests that carbon-nitrogen allocation became more closely linked to water-use efficiency under stronger water stress [46,47,48]. Overall, the multidimensional regulation of WUE_i_ in *P. euphratica* shifted from a pattern primarily driven by leaf carbon investment to one dominated by the coordinated optimization of carbon–nitrogen allocation and leaf anatomical traits, enabling adaptation to the stronger water stress associated with deeper groundwater. Although this study clarified the multidimensional adjustment of *P. euphratica* across different groundwater depths, these conclusions are based primarily on observational data from typical stands within the study area. Because desert riparian forests are characterized by strong spatial heterogeneity, and long-term physiological and morphological responses may be jointly influenced by stand age structure and local microenvironmental conditions, the trait trade-off thresholds identified here may have certain regional limitations. Future studies should therefore incorporate greater sampling intensity to evaluate the broader applicability of these findings.

## 4. Materials and Methods

### 4.1. Study Area Description

**Figure 8:**
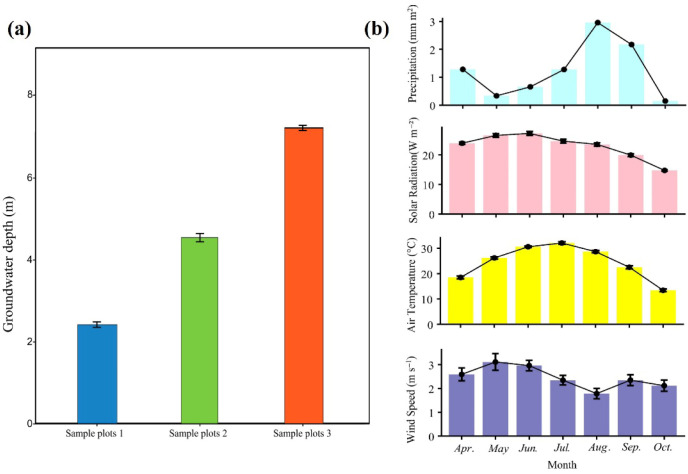
Variations in groundwater depth among different sampling plots and trends in meteorological factors in the study area from April to October. (**a**) Mean groundwater depth at the three plots; (**b**) From top to bottom is monthly total precipitation, monthly mean solar radiation, monthly mean air temperature, monthly mean wind speed from April to October, respectively. Plots 1, 2, and 3 correspond to groundwater depths of 2.19, 4.88, and 7.45 m, respectively, and are denoted throughout this paper as “2 m”, “5 m”, and “7 m”.

The study area is situated within the desert riparian forest along the middle reaches of the Tarim River (84°15′~85°30′ E, 40°55′~41°15′ N), China. The region has a warm temperate continental arid desert climate [49]. The mean annual temperature is approximately 9.7~10.8 °C, with the coldest month being January (−10~0 °C) and the warmest month being July (20~30 °C) [50], mean annual precipitation is typically below 50 mm and potential evapotranspiration greatly exceeds precipitation (1887~2910 mm). The elevation ranges from 800 to 940 m above sea level [51]. Soil types are predominantly alluvial, formed by floodplain and river channel deposits, commonly including alluvial meadow soils and soils of varying salinity [52]. The dominant arboreal species is *Populus euphratica*. Common shrub species include *Tamarix chinensis* Lour., *Alhagi camelorum* Fisch. and *Lycium ruthenicum* Murray. Dominant herbaceous species include *Glycyrrhiza uralensis* Fisch. and *Bassia scoparia* (L.) A. J. Scott [53]. Meteorological data shown in Figure 8 were obtained from the Xinjiang Field Scientific Observation and Research Station of Luntai *Populus euphratica* Forest Ecosystem for the period April to October 2025, including monthly precipitation, solar radiation, air temperature, and wind speed. The meteorological station is located approximately 12 km from the study plots. Specific environmental conditions during the growing season are shown in Figure 8.

This study was conducted in natural *P. euphratica* forests in the middle reaches of the Tarim River from April to October. Three plots were selected with stable groundwater depths of approximately 2.19, 4.88, and 7.45 m respectively. Groundwater depth of each plot was measured using buried observation wells. The values reported represent growing-season means (April–October), and seasonal fluctuations were less than ±0.5 m at all three plots, representing shallow, middle, and deep groundwater scenarios, respectively. All three plots were located on river terraces with flat terrain, similar soil types, and *P. euphratica* as the dominant vegetation. No significant human disturbances such as logging, grazing, or construction were observed, minimizing environmental differences other than groundwater depth. Within each plot, a 20 m × 20 m quadrat was delineated. Four mature *P. euphratica* trees with similar diameter at breast height (ca. 25 cm) and tree height (ca. 8 m), free from obvious pests, diseases, and mechanical damage, and with intact crowns and good vigor, were randomly selected from each quadrat. The distance between adjacent trees was no less than 5 m to reduce spatial autocorrelation. Leaf and branch samples were collected from each tree according to uniform protocols, and various functional traits were measured.

### 4.2. Gas Exchange and Chlorophyll Fluorescence Measurements

Gas exchange parameters were measured on clear mornings between 8:00 and 12:00. Branches of similar size were excised from the sun-exposed side of selected *P. euphratica* trees in each plot, at a height of approximately 6 m. These branches were immediately placed in a water bucket for ex situ measurements, with the entire process completed within 20 min to minimize excision stress [54,55]. The following gas exchange parameters were determined: transpiration rate (Tr, mmol m^−2^ s^−1^), intercellular to ambient CO_2_ concentration ratio (Ci/Ca), net photosynthetic rate (P_n_, μmol m^−2^ s^−1^), and stomatal conductance (gs, mol m^−2^ s^−1^). Measurements were performed using a LI-6800 portable photosynthesis system (Li-Cor, Lincoln, NE, USA). During measurements, the leaf chamber was maintained at a CO_2_ concentration of 400 μmol mol^−1^, a photosynthetic photon flux density (PPFD) of 1500 μmol m^−2^ s^−1^, a leaf temperature of 25 °C, and a relative humidity of 60%.

Chlorophyll fluorescence parameters, specifically the maximum quantum yield of PSII (Fv/Fm), were measured on dark-adapted leaves using the same LI-6800 system. Intact leaves on selected branches were wrapped in aluminum foil clips for 30 min to achieve dark adaptation; fluorescence measurements were then performed immediately on the same branches used for gas exchange, within 2 h of excision. For all parameters, three replicate measurements were taken per tree (one measurement per leaf on three leaves), and the mean value was used.

### 4.3. Morphological and Hydraulic Trait Measurements

Stomatal traits were determined on mature, sun-exposed leaves consistent with those used for gas exchange measurements. Samples were collected using the nail polish impression method [56,57]. A thin, even layer of clear nail polish was applied to the mid-abaxial surface of each leaf, avoiding the main veins and leaf margins. After drying at room temperature for 10 min, the impression film was peeled off with transparent adhesive tape and mounted onto a glass slide to create a temporary microscopic preparation. Observations and photographic documentation were performed using an Olympus CX33 biological microscope (Olympus Corp., Tokyo, Japan). Stomatal density (SD, pores mm^−2^) was calculated as the ratio of stomatal number to the image area (excluding veins). Stomatal size (StS, μm^2^) and stomatal aperture were also determined. For each individual, five leaves were sampled, and five vein-free fields of view were randomly selected from each leaf for measurement, after which the average value was calculated. Image processing and analysis were conducted using Image-Pro Plus (Media Cybernetics, Rockville, MD, USA). Stomatal size and stomatal area fraction were calculated as in Equations (1) and (2):

(1) $$StS=\frac{\pi }{4}\times S_{L}\times S_{W}$$

(2) $$SAF(\%)=SD\times StS\times 10^{-4}$$

where $S_{L}$, $S_{W}$, and *SAF* denote stomatal length, stomatal width, and stomatal area fraction, respectively.

Leaf and branch anatomy was determined using the conventional paraffin sectioning method following standard plant tissue preparation techniques [58]. Pre-fixed samples (fixed for ≥24 h) were subjected to a graded ethanol dehydration series, followed by clearing, wax infiltration, and paraffin embedding. Continuous sections with a thickness of 8 μm were cut using a rotary microtome. Sections were double-stained with Safranin-Fast Green: immersion in a 1% Safranin aqueous solution for 12 h, followed by counterstaining with 0.5% Fast Green. After clearing with xylene, sections were mounted with neutral balsam [59]. Observations were made under an Olympus CX33 microscope, and representative sections were photographed and documented. Images were analyzed quantitatively using Image-Pro Plus. Measured traits included leaf thickness (LT), mesophyll thickness (MT), cuticle thickness (LCT), epidermal thickness (LET), and vein number per unit leaf length (Nvll). For each sample, five fields of view were randomly selected for measurement, and the mean value was used for subsequent analysis. Specifically, LT and MT were each measured at five positions per section under 50× magnification and averaged; LCT and LET were each measured at five positions under 200× magnification and averaged. Vein number was counted under 100× magnification, and Nvll was calculated as vein number divided by the corresponding leaf length.

Stem hydraulic conductivity was measured using a XYL’EM-Plus embolism measurement system (Bronkhorst, Montigny-les-Cormeilles, France) [60]. A stable pressure head (≈0.007 MPa) was provided by a ~70 cm water column. Freshly collected branches were defoliated. To prevent refilling of embolized conduits and ensure clean cut surfaces, each end of the branch segment was re-cut by 1–2 cm under degassed deionized water. The segment was then connected to the measurement circuit. The initial hydraulic conductivity (K_0_; kg s^−1^ MPa^−1^) was automatically recorded by the system under steady-state flow.

Subsequently, the sample was connected to a flushing circuit. Degassed deionized water was flushed through the segment at 0.2 MPa until the flow rate stabilized, as indicated by a variation of less than 5% within a 1 min interval (typically ~20 min). The flushing duration was determined by real-time monitoring of the flow rate via computer; embolism was considered effectively removed when the flow rate no longer increased. The sample was then returned to the measurement circuit to obtain the maximum hydraulic conductivity (K_max_; kg s^−1^ MPa^−1^). For each tree, measurements were performed on three branches of similar size, and the mean was used as the v individual-level value (totaling 12 branches per plot and 36 branches across all three plots). Sapwood-specific hydraulic conductivity (K_s_) and percentage loss of conductivity (PLC) were calculated according to Equations (3) and (4) [61].

(3) $$K_{s}=\frac{K_{0}\times L}{S}$$

(4) $$PLC=\left(1-\frac{K_{0}}{K_{max}}\right)\times 100\%$$

where *L* represents the sample length (m) and *S* represents the stem cross-sectional area (m^2^).

A 3–5 cm segment was excised from the branches previously used for hydraulic conductivity measurements. After removing the bark, the segment was immersed in distilled water, and its volume was determined using Archimedes’ principle. The segment was then oven-dried at 75 °C for 72 h, and its dry mass was measured. Wood density (WD) was calculated as in Equation (5):

(5) $$WD(g\;{cm}^{-3})=\frac{D_{stem}}{V_{stem}}$$

where *D_stem_* is the dry mass of the excised stem segment, and *V_stem_* is the volume determined by water displacement.

The branches used for measuring leaf photosynthetic traits were identified. Their inner diameter was measured after removing the bark and pith, to calculate the sapwood area (SA, mm^2^). All leaves were then excised from these branches. Leaf area (LA, cm^2^) was measured at the field station using an EPSON scanner (Seiko Epson Corporation, Suwa, Nagano, Japan) and Image-Pro Plus software (https://mediacy.com/image-pro/version-comparison/, accessed on 15 March 2026). Leaf fresh mass (W_F_, g) was first determined. Leaves were then blanched in an oven at 105 °C for 30 min, followed by drying at 75 °C for 72 h to obtain leaf dry mass (W_D_, g). The Huber value (HV) was calculated as in Equation (6):

(6) $$HV\left({mm}^{2}cm^{-2}\right)=\frac{SA}{LA}$$

Based on *W_F_*, *W_D_*, *LA*, specific leaf mass (SLM; g m^−2^), leaf water content (LWC; %), and leaf dry matter content (LDMC; %) were calculated according to Equations (7)–(9):

(7) $$SLM=\frac{W_{D}}{LA}$$

(8) $$LWC=\frac{W_{F}-W_{D}}{W_{F}}$$

(9) $$LDWC=\frac{W_{D}}{W_{F}}$$

For chlorophyll content determination, fresh leaves collected from the branches described, above were immersed in an acetone: Tris buffer solution (80:20, *v*/*v*) at 4 °C, sealed and kept in the dark for over 48 h for extraction, after which absorbance (Ab) was measured at wavelengths of 470, 537, 647, and 663 nm using a Thermo Scientific GENESYS 50 UV-Visible spectrophotometer (Thermo Fisher Scientific, Waltham, MA, USA), and pigment concentrations were calculated following Sims and Gamon [62].

### 4.4. Measurement of Leaf Nutrients

Dried leaf samples were ground into a fine powder for subsequent analysis. Leaf organic carbon (LCC) was determined using the potassium dichromate-external heating method [63]. Total nitrogen (TN) and total phosphorus (TP) were determined by acid digestion followed by analysis on an AA3 continuous flow analyzer (Bran + Luebbe, AAIII, Norderstedt, Germany) [64].

### 4.5. Measurement of Leaf δ^13^C and Long-Term Intrinsic Water-Use Efficiency

Ground and stored samples were further milled to pass a 100-mesh sieve. A 5–8 mg subsample of each sample was weighed, and the ^13^C/^12^C ratio was determined using a continuous-flow elemental analyzer-isotope ratio mass spectrometer (EA-IRMS) system, consisting of an EA3028 elemental analyzer (EuroVector, Pavia, Italy) coupled with a Perspective stable isotope ratio mass spectrometer (Nu Instruments, Wrexham, UK). Results were expressed as δ^13^C (‰) relative to the Vienna Pee Dee Belemnite (VPDB) standard (R = 0.01124). Leaf carbon isotope discrimination (Δ^13^C) was calculated using the δ^13^C of atmospheric CO_2_ (δ^13^C_air_ = −8‰) and the δ^13^C of the leaf (δ^13^C_leaf_). Long-term intrinsic water-use efficiency (WUE_i_; μmol mmol^−1^) was estimated from Δ^13^C based on the C_3_ plant leaf-scale carbon isotope discrimination model [65,66,67,68]. The CO_2_ compensation point in the absence of mitochondrial respiration (Γ*) was calculated as a function of leaf temperature (T_leaf_) according to Equations (10)–(13).

(10) $$\delta^{13}C_{leaf}=\left(\frac{R_{sample}}{R_{standard}}-1\right)\times 1000‰$$

(11) $${∆}^{13}C=\frac{\delta^{13}C_{air}-\delta^{13}C_{leaf}}{1+\delta^{13}C_{leaf}/1000}$$

(12) $$\Gamma^{*}=42.7+1.68\left(T_{leaf}-25\right)+0.012{\left(T_{leaf}-25\right)}^{2}$$

(13) $$WUE_{i}=\frac{C_{a}}{1.6}\times \frac{b-{∆}^{13}C-f^{\prime }\times \frac{\Gamma^{*}}{C_{a}}}{b-a_{s}+\frac{g_{s}}{g_{m}}\times (b-a_{m})}$$

where Ca is the atmospheric CO_2_ concentration (400 ppm); a_s_ is the stomatal discrimination factor (4.4‰); a_m_ is the mesophyll discrimination factor (1.8‰); *b* is the Rubisco carboxylation fractionation factor (29‰); $f^{’}\;$ is the photorespiration-related fractionation term (11.2‰); and *T_leaf_* is the leaf temperature (25 °C). The ratio of stomatal conductance to CO_2_ (g_s_) to mesophyll conductance (g_m_) was taken as 0.71 for deciduous trees [66].

### 4.6. Statistical Analysis

All statistical analyses were performed in R (version 4.5.1; R Core Team, Vienna, Austria, 2025). Prior to analysis, normality was assessed using the Shapiro–Wilk test and homogeneity of variance was verified using Levene’s test. Data meeting these criteria were compared using one-way ANOVA with LSD post hoc tests. Each groundwater depth was replicated with *n* = 4 trees, with three measurements per tree averaged to give one value per individual. All traits were first standardized using Z-scores. Before PCA, variables highly susceptible to instantaneous environmental fluctuations, (e.g., gas exchange and instantaneous hydraulic indices), as well as highly collinear or functionally redundant variables, were removed. Sixteen relatively stable and representative traits, spanning leaf morphology, stomatal and anatomical structure, leaf economics and nutrient status, photosynthetic performance, and long-term water-use efficiency were retained. The first two principal components and their loadings were extracted from the standardized matrix (using the prcomp function in the ‘stats’ package). Euclidean distance matrices between WUE_i_ and each functional trait were constructed for each groundwater depth, and distance correlations were evaluated using Mantel test (the ‘vegan’ package); Mantel’s *r* was used to characterize coupling strength, and the *p*-value from a permutation test (999 permutations) was used to determine significance). Pearson correlation coefficients between WUE_i_ and traits within the same groundwater depth were also calculated. Figures were produced using the ‘ggplot2’ and ‘ggcor’ packages. Data are presented as mean ± standard deviation.

## 5. Conclusions

This study provides a comprehensive understanding of the coordinated adaptive mechanisms of *P. euphratica* functional traits along a groundwater depth gradient. As groundwater depth increased, leaf P_n_ and Fv/Fm declined significantly, whereas long-term intrinsic water use efficiency increased substantially, supporting hypothesis H1 that groundwater drawdown forces a shift from carbon acquisition toward water conservation. These changes reflected a multidimensional adaptive strategy: (1) significant reductions in stomatal size, stomatal area fraction, and leaf thickness limited transpirational water loss, supporting hypothesis H2 regarding stomatal–anatomical coordination; (2) increased PLC and Huber value, together with decreased Ks, indicated a shift toward a more conservative hydraulic strategy; (3) decreased leaf water content, coupled with significant increases in specific leaf mass, LDMC, and leaf nitrogen and phosphorus, enhanced resource-use efficiency under water limitation, consistent with the concentration-effect mechanism proposed in hypothesis H3. *P. euphratica* increased water-use efficiency through a shift from leaf carbon investment to the coordinated adjustment of carbon–nitrogen allocation and leaf anatomical traits. These findings provide valuable insights for regional vegetation restoration and desertification control, offering scientific support for maintaining the long-term ecological functions of desert vegetation.

## Figures and Tables

**Table 1:** Leaf morphological and structural characteristics and chlorophyll content of *Populus euphratica* at different groundwater depths. Chla, chlorophyll *a* (mg g^−1^); Chlb, chlorophyll *b* (mg g^−1^); Chla/Chlb, chlorophyll *a*/*b* ratio; SLM, specific leaf mass (g m^−2^); LWC, leaf water content (%); LDMC, leaf dry matter content (%). Different lowercase letters denote significant differences among groundwater depths (one-way ANOVA with LSD post hoc test, *p* < 0.05). Values are means ± SD (*n* = 4).

Depth	Chla	Chlb	Chla/Chlb	SLM	LWC	LDMC
2 m	1.15 ± 0.02 a	0.29 ± 0.01 a	3.95 ± 0.12 b	129.90 ± 13.02 b	65.86 ± 1.68 a	34.14 ± 1.65 c
5 m	0.71 ± 0.01 b	0.16 ± 0.01 b	4.32 ± 0.05 a	125.18 ± 11.51 b	60.78 ± 6.66 b	39.23 ± 6.67 b
7 m	0.76 ± 0.16 b	0.20 ± 0.03 b	3.75 ± 0.20 b	135.90 ± 11.57 a	57.54 ± 4.72 c	43.19 ± 4.00 a

## Data Availability

The original contributions presented in this study are included in the article. Further inquiries can be directed to the corresponding author.

## Abbreviations

The following abbreviations are used in this manuscript.

Specific leaf mass	SLM	g·m^−2^
Leaf water content	LWC	%
Leaf dry matter content	LDMC	%
Transpiration rate	T_r_	mmol m^−2^ s^−1^
Stomatal conductance	g_s_	mol m^−2^ s^−1^
Net photosynthetic rate	P_n_	μmol m^−2^ s^−1^
Long-term intrinsic water-use efficiency	WUE_i_	μmol mmol^−1^
Intercellular carbon dioxide concentration/Atmospheric carbon dioxide concentration	Ci/Ca	—
Maximum quantum yield of PSII	F_v_/F_m_	—
Stomatal size	StS	μm^2^
Stomatal density	SD	pore mm^−2^
Stomatal area fraction	SAF	%
Leaf organic carbon	LCC	g kg^−1^
Leaf total nitrogen	TN	g kg^−1^
Leaf total phosphorus	TP	g kg^−1^
Organic carbon/Total nitrogen	C/N	—
Organic carbon/Total phosphorus	C/P	—
Total nitrogen/Total phosphorus	N/P	—
Leaf thickness	LT	mm
Mesophyll thickness	MT	mm
Mesophyll conductance	g_m_	mol m^−2^ s^−1^
Leaf cuticle thickness	LCT	mm
Leaf epidermal thickness	LET	mm
Number of veins per unit leaf length	Nvll	mm^−1^
Chlorophyll	Chl	mg g^−1^
Chlorophyll a	Chla	mg g^−1^
Chlorophyll b	Chlb	mg g^−1^
Chlorophyll a/Chlorophyll b	Chla/Chlb	—
Huber value	HV	mm^2^ cm^−2^
Percentage loss of hydraulic conductivity	PLC	%
Specific hydraulic conductivity	K_s_	kg s^−1^ m^−2^ MPa^−1^
Wood density	WD	g cm^−3^
